# Minimally Invasive Management of Recurrent Craniopharyngioma: Intraoperative Real-Time Magnetic Resonance Imaging-Guided Transnasal Cyst Aspiration—A Case-Based Technical Note

**DOI:** 10.1055/a-2753-9498

**Published:** 2025-12-18

**Authors:** Oyku Ozturk, Emetullah Cindil, Metin E. Demirkan, Muammer M. Sahin, Emrah Celtikci

**Affiliations:** 1 Department of Neurosurgery, Gazi University Faculty of Medicine, AnkaraTurkey; 2 Department of Radiology, Gazi University Faculty of Medicine, AnkaraTurkey; 3 Department of Otorhinolaryngology/Head and Neck Surgery, Gazi University Faculty of Medicine, AnkaraTurkey

**Keywords:** craniopharyngioma, cyst aspiration, intraoperative MRI, neuronavigation, skull base surgery

## Abstract

**Objective:**

Craniopharyngiomas are rare benign epithelial tumors typically located in the sellar and suprasellar region. Standard management includes surgery and radiotherapy. However, recurrence in the form of isolated cystic lesions presents a unique challenge. Herein, we report a case of recurrent cystic craniopharyngioma treated using intraoperative real-time magnetic resonance imaging (MRI) and neuronavigation-guided transnasal aspiration.

**Methods:**

The patient was evaluated with MRI, including T1- and T2-weighted sequences with contrast enhancement. A transnasal surgical approach was planned using neuronavigation. Intraoperative real-time MRI was used to guide catheter placement and ensure complete cyst aspiration. The procedure was performed under general anesthesia using standard sterile technique, and histopathological examination of the aspirated material was conducted.

**Results:**

A 77-year-old female previously underwent endoscopic transnasal resection for a cystic craniopharyngioma confirmed to be of the adamantinomatous subtype. During follow-up, a recurrent suprasellar cystic lesion was identified. The cyst was aspirated intraoperatively via an external ventricular drainage catheter placed through the transnasal route. Real-time MRI using fast low-angle shot (FLASH) sequences enabled real-time monitoring and confirmation of cyst reduction.

**Conclusion:**

This case illustrates a minimally invasive, safe, and effective technique for managing recurrent cystic craniopharyngiomas using real-time MRI guidance. It highlights the potential of image-guided aspiration as a first-line approach in select patients, especially where conventional surgical reintervention may pose significant risks.

## Objective

Craniopharyngiomas are epithelial tumors derived from remnants of Rathke's pouch, accounting for less than 5% of all intracranial neoplasms. 1 2 3 They are subclassified into adamantinomatous and papillary types, each with distinct histopathological and demographic profiles. 4 The adamantinomatous type, which exhibits bimodal age distribution and a tendency for cyst formation and calcification, is more commonly seen in pediatric and older adult populations. 3 5

While complete surgical resection is often pursued, recurrence is frequent, particularly in cases where subtotal resection is performed to preserve neurovascular structures. Cystic recurrence may cause compressive symptoms and visual deterioration. In such scenarios, minimally invasive strategies—especially those enhanced by real-time imaging—may provide effective intervention with reduced morbidity.

This report presents a case of recurrent solitary cystic craniopharyngioma in an elderly patient successfully managed via real-time magnetic resonance imaging (MRI)-guided transnasal aspiration. The case emphasizes the utility of intraoperative imaging in guiding targeted neurosurgical procedures.

## Methods

The patient was evaluated with MRI, including T1- and T2-weighted sequences with contrast enhancement. A neuronavigation system was used for planning the transnasal surgical approach. Intraoperative real-time MRI (T2-weighted fast low-angle shot [FLASH] sequences) was applied to monitor catheter placement and confirm complete aspiration of the cyst contents. The procedure was performed under general anesthesia using standard sterile technique. Histopathological examination of the aspirated material was conducted. All procedures were performed in accordance with institutional ethical standards, and informed consent was obtained from the patient.

## Results

A 77-year-old woman presented with progressive visual decline. Initial MRI revealed a sellar–suprasellar cystic mass with contrast enhancement. She underwent endoscopic transnasal transsphenoidal resection. Histopathology confirmed an adamantinomatous craniopharyngioma ( Fig. 1 ).

**Fig. 1:**
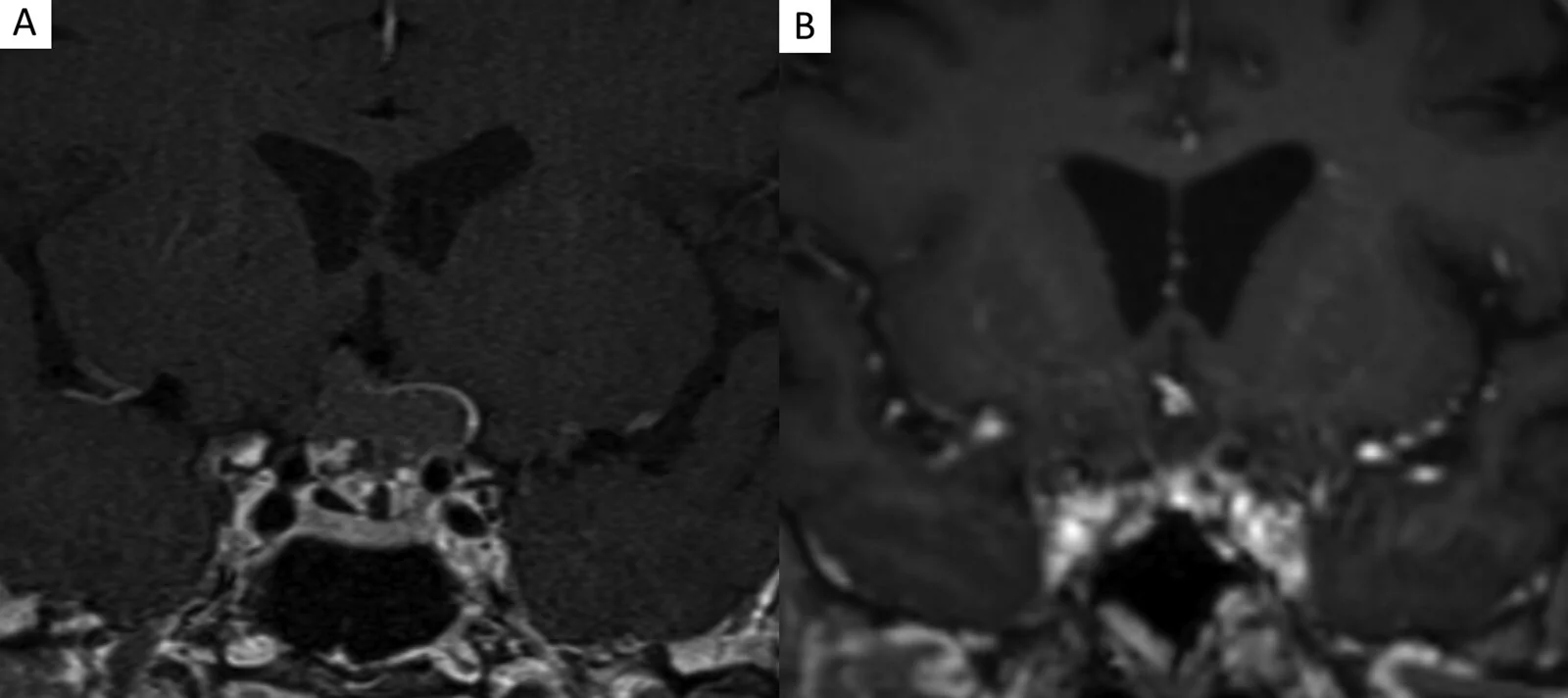
Preoperative coronal ( **A** ) T1-weighted postcontrast MRI showing a cystic suprasellar mass. Postoperative image ( **B** ) after initial resection reveals residual fibrosis. MRI, magnetic resonance imaging.

Follow-up imaging revealed a recurrent cystic lesion in the suprasellar region ( Fig. 2 ). Given the patient's age and prior surgery, a minimally invasive approach was favored. Under general anesthesia, a drainage catheter was inserted transnasally under neuronavigation. Real-time MRI guidance (FLASH T2-weighted sequences) enabled visualization of the catheter and continuous monitoring of the aspiration process ( Fig. 3 ).

**Fig. 2:**
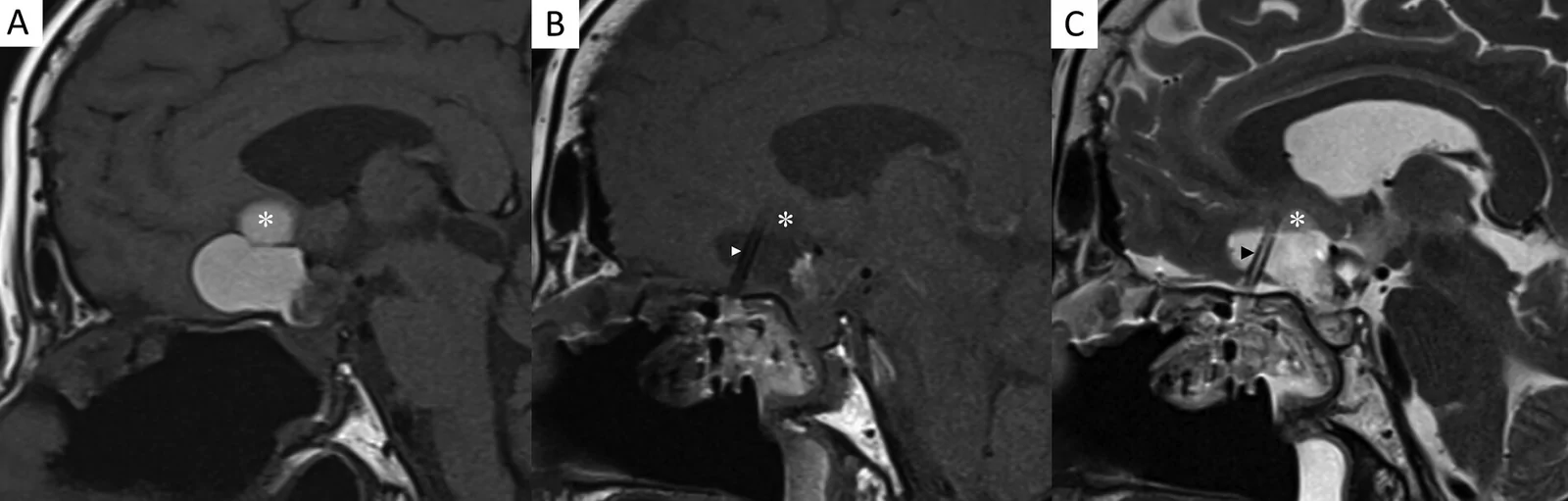
Sagittal T1-weighted ( **A, B** ) and T2-weighted ( **C** ) MRI, demonstrating the recurrent cyst (white and black arrowhead) before and after aspiration. Drainage catheter visible within the lesion; cyst volume markedly reduced. MRI, magnetic resonance imaging.

**Fig. 3:**
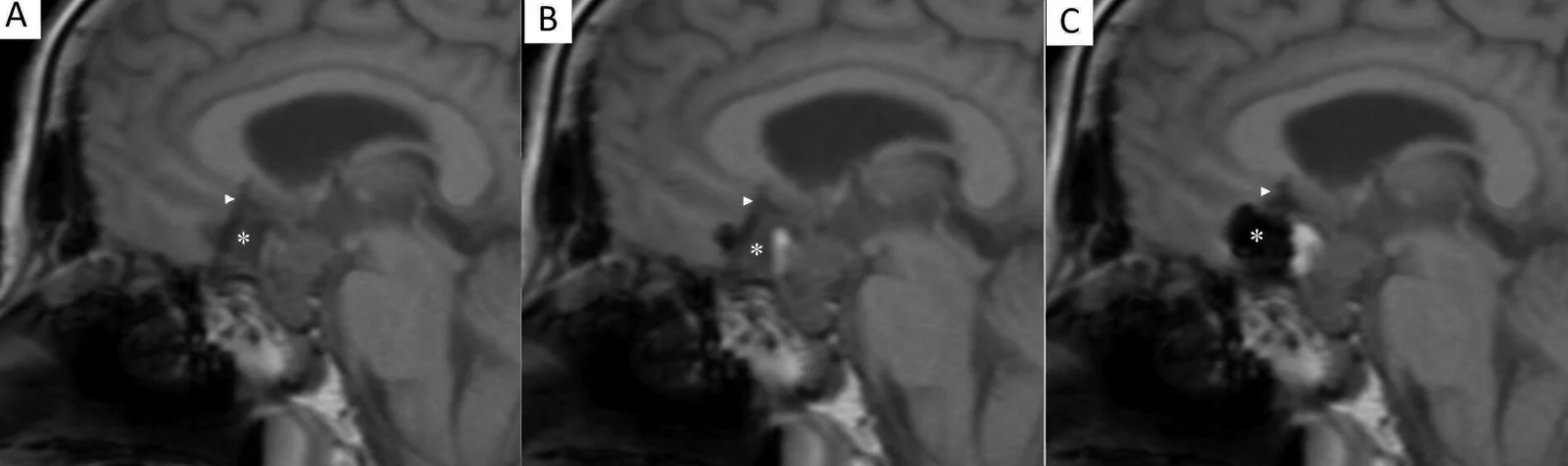
Sequential FLASH images during aspiration via drainage catheter (white arrowhead) showing dynamic reduction in cyst size and increasing air space (white asterisk), indicating successful decompression. FLASH, fast low-angle shot.

The cyst contents, consistent with cerebrospinal fluid and devoid of atypical cells, were aspirated. Imaging confirmed a marked reduction in cyst volume. Postoperative recovery was uneventful, with improvement in visual symptoms.

## Discussion

MRI-guided interventions are increasingly utilized in neurosurgery, particularly for biopsies, drainage procedures, and lesion localization. Intraoperative real-time imaging not only improves surgical accuracy but also reduces the need for extensive exposure and manipulation.

In the context of craniopharyngioma recurrence, especially with isolated cystic components, reoperation poses a significant risk due to previous scarring and anatomical distortion. In such settings, catheter-based aspiration under real-time MRI offers distinct advantages; precise localization of cyst and catheter tip, real-time confirmation of decompression, and minimization of invasiveness, especially in elderly or comorbid patients. 6 7 8 9 10

Our experience supports the application of this method in selected cases. While broader adoption may be limited by MRI access and cost, centers equipped with intraoperative MRI capability should consider this as a first-line option for recurrent cystic lesions.

## Conclusion

Intraoperative MRI-guided aspiration of recurrent craniopharyngioma cysts is a safe, effective, and minimally invasive alternative to conventional reoperation. It enables dynamic visualization, ensures precise intervention, and reduces surgical morbidity—particularly beneficial in elderly patients or those with high surgical risk.
